# Evaluation of energy metabolism and calcium homeostasis in cells affected by Shwachman-Diamond syndrome

**DOI:** 10.1038/srep25441

**Published:** 2016-05-05

**Authors:** Silvia Ravera, Carlo Dufour, Simone Cesaro, Roberta Bottega, Michela Faleschini, Paola Cuccarolo, Fabio Corsolini, Cesare Usai, Marta Columbaro, Marco Cipolli, Anna Savoia, Paolo Degan, Enrico Cappelli

**Affiliations:** 1 DIFAR-Biochemistry Lab., Department of Pharmacy, University of Genova, 16132 Genova, Italy; 2 Haematology Unit, Istituto Giannina Gaslini, 16148 Genova, Italy; 3 Oncoematologia Pediatrica, Azienda Ospedaleira universitaria Integrata, Verona, Italy; 4 Institute for Maternal and Child Health – IRCCS Burlo Garofolo, Trieste, Italy; 5 S. C. Mutagenesis, IRCCS AOU San Martino – IST (Istituto Nazionale per la Ricerca sul Cancro), CBA Torre A2, 16123 Genova, Italy; 6 Centro Diagnostica Genetica e Biochimica Malattie Metaboliche, Istituto Giannina Gaslini, 16148 Genova, Italy; 7 Institute of Biophysics, National Research Council, 16149 Genova, Italy; 8 SC Laboratory of Musculoskeletal Cell Biology, IOR, Bologna, Italy; 9 Cystic Fibrosis Centre, Azienda Ospedaliera Universitaria, Piazzale Stefani, 1-37126 Verona, Italy; 10 Department of Medical Sciences, University of Trieste, Trieste, Italy

## Abstract

Isomorphic mutation of the SBDS gene causes Shwachman-Diamond syndrome (SDS). SDS is a rare genetic bone marrow failure and cancer predisposition syndrome. SDS cells have ribosome biogenesis and their protein synthesis altered, which are two high-energy consuming cellular processes. The reported changes in reactive oxygen species production, endoplasmic reticulum stress response and reduced mitochondrial functionality suggest an energy production defect in SDS cells. In our work, we have demonstrated that SDS cells display a Complex IV activity impairment, which causes an oxidative phosphorylation metabolism defect, with a consequent decrease in ATP production. These data were confirmed by an increased glycolytic rate, which compensated for the energetic stress. Moreover, the signalling pathways involved in glycolysis activation also appeared more activated; i.e. we reported AMP-activated protein kinase hyper-phosphorylation. Notably, we also observed an increase in a mammalian target of rapamycin phosphorylation and high intracellular calcium concentration levels ([Ca^2+^]_i_), which probably represent new biochemical equilibrium modulation in SDS cells. Finally, the SDS cell response to leucine (Leu) was investigated, suggesting its possible use as a therapeutic adjuvant to be tested in clinical trials.

Shwachman–Diamond syndrome (SDS) is an autosomal recessive disease characterized by pancreatic insufficiency, skeletal abnormalities, bone marrow failure and predispositions to myelodysplastic syndrome (MDS) and acute myeloid leukaemia (AML)^1^,^2^. SDS is caused by mutations in the *SBDS* (Shwachman-Bodian-Diamond syndrome) gene, which account for approximately 90% of affected individuals^3^. Consistent with the hypothesis that at least one *SBDS* variant is hypomorphic, knock-out *Sbds*−/− mice are embryonic lethal^4^.

The SBDS protein localizes in the nucleolus where it plays an important role in ribosome biogenesis, as was shown in several studies^5^,^6^,^7^. SBDS is also involved in other molecular processes that may be independent of ribosome biogenesis, such as chemotaxis^8^, mitotic spindle formation^9^ and cellular stress responses. Regarding the latter aspect, in SBDS depleted human cells, endoplasmic reticulum (ER) stress is associated with caspase 3 cleavage and activation of the intrinsic apoptotic pathway, as well as eukaryotic initiation factor 2-alpha (eIF2-alpha) phosphorylation, which rapidly reduces mRNA translation initiation^10^. Finally, the silencing of the *SBDS* gene in human cells and a yeast model depleted of its SBDS orthologue (Sdo1) display reduced mitochondrial functionality^11^. In turn, ER and mitochondrial stress may induce reactive oxygen species (ROS) production leading to increased apoptosis and decreased cell growth^12^. Ribosome biogenesis and mRNA translation are high energy-demanding processes.

The aim of this study was to characterize the energetic and respiratory profiles and the biochemical pathways that may modulate these processes in SDS cells.

## Results

### Energetic metabolism is defective in SDS cells

Bone marrow failure with leukaemic evolution is a clinical feature not only of SDS but also of other inherited marrow failure diseases, such as Fanconi anaemia. As Fanconi anaemia cells have defective energetic metabolism because of reduced respiration and ATP production rates^13^,^14^,^15^ and as ribosome biogenesis and mRNA translation require high amounts of energy, we investigated energetic metabolism in SDS cells.

**Figure 1:**
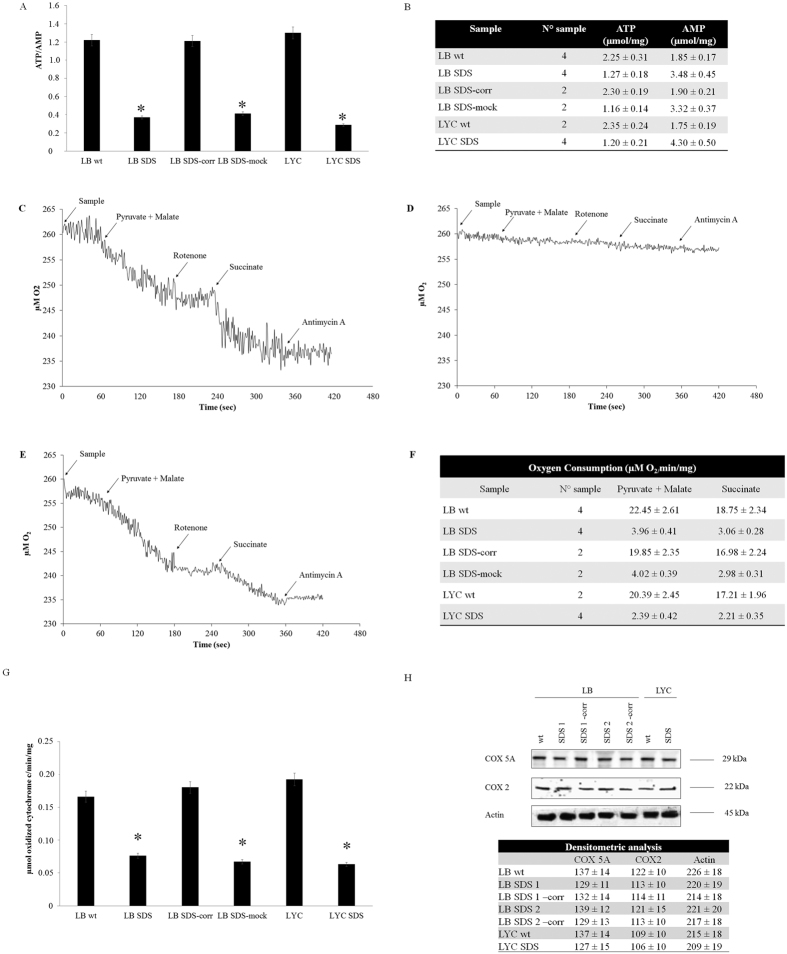
Energetic metabolism is defective in SDS cells. (**A**) The AMP/ATP ratio was calculated using data from the table in (**B**) (*P < 0.001). (**B**) The ATP and AMP contents measured in lymphoblasts (LB) and lymphocytes (LYC) of wt, SDS and their isogenically corrected counterparts (SDS-corr) cells. The AMP and ATP assays routinely employed cell homogenates from 100 to 200,000 cells. The data are expressed as means ± SD of at least 3 different experiments. Panels (**C–E**) show the oxygen consumption in wt, SDS and SDS-corr cells measured with an amperometric electrode, respectively. Panel (**F**) shows the number of samples examined and the oxygen consumption values (μM O2/min/mg) at the level of complex I (pyruvate/malate) or complex II (succinate) of oxidative phosphorylation. The data are expressed as means ± SD of at least 3 different experiments. Two-hundred and fifty to 500,000 cells were routinely analysed for each oxymetric titration. (**G**) The complex IV (cytochrome c oxidase) activity was measured following oxidation of ascorbate-reduced cytochrome C at 550 nm. The data were obtained from the medians of at least 3 different experiments. *p < 0.001. (**H**) Western blots of COX5A and COX2, which are complex IV components of nuclear and mitochondrial DNA origin, respectively. SDS and wt lymphoblast and lymphocyte extracts showed comparable levels of both proteins, as demonstrated by the densitometric analysis reported in the table (the data are reported as the relative optical density normalized to the actin signal). Legend: LB (lymphoblasts), LYC (lymphocytes), SDS (Shwachman-Diamond syndrome), wt (wild type), SDS-corr (Shwachman-Diamond syndrome transfected with SBDS gene), SDS-mock (Shwachman-Diamond syndrome transfected with empty vector).

First, we measured the intracellular concentrations of ATP and AMP as expressed by the ATP/AMP ratio. As reported in Fig. 1A, the ATP/AMP ratio was significantly lower in the SDS cells than in the controls, both in primary lymphocytes (LYC) and in lymphoblast (LB) cell lines. In particular, we observed a reduction in ATP and an accumulation of AMP concentrations, suggesting a strong deficit in energy production (Fig. 1B). Indeed, expression of the wild-type (wt) form of *SBDS* in mutant lymphoblast cells restored the ATP/AMP ratio, suggesting a role for SBDS protein in energy production (Fig. 1B).

The principal source of cellular ATP is oxidative phosphorylation (OXPHOS). This system produces energy by oxygen consumption only when the respiratory complex activity is coupled with functioning FoF1 ATP synthase^16^. By contrast, in uncoupled conditions, where the respiring complexes are uncoupled from ATP synthase, oxygen consumption is not associated with the ATP production, which can induce oxidative stress^17^. To evaluate oxidative metabolism, we then measured oxygen consumption after stimulation of the respiratory pathways using pyruvate/malate or succinate, which are substrates linked to the activity of complexes I, III and IV and complexes II, III and IV, respectively. In control cells, the two substrates induced increased oxygen consumption (Fig. 1C), while in the SDS cells, oxygen consumption was significantly lower and the decrease was more evident with the pyruvate/malate substrate (Fig. 1D). These findings were consistent with a lower ATP/AMP ratio and indicate that in SBDS cells, OXPHOS activity is impaired. Similar to the ATP/AMP ratio, oxygen consumption was restored to normal in complemented SDS cells (Fig. 1E), which further supported the involvement of SBDS protein in energy metabolism and mitochondrial function.

Considering that the defective respiratory pathways share complexes III and IV, we hypothesized that the activity of one of the two complexes was impaired in SDS. Although complex III activity was similar to the control (Supplementary Figure 2C), complex IV activity was dramatically lower in the SDS cells (Fig. 1G), while it was restored to normal in the complemented SDS cells. In support of this finding, complex I and II activities were in the normal range (Supplementary Figure 2A,B) in the control cells.

Complex IV is made up of 32 proteins that are encoded by nuclear or mitochondrial DNA. To investigate if complex IV deficiency was due to reduced expression of these proteins, we chose to assess the expression levels of two components of complex IV, COX5A and COX2, which are codified by nuclear and mitochondrial DNA, respectively. As shown in Fig. 1H, COX5A and COX2 were expressed at normal levels in the SDS cells, which suggests that a functional deficiency of complex IV did not seem to be due to defective protein expression. Moreover, the restoration to normal complex IV function observed after complementation of the SDS cells suggests that this defect was related to faulty SBDS proteins.

**Figure 2:**
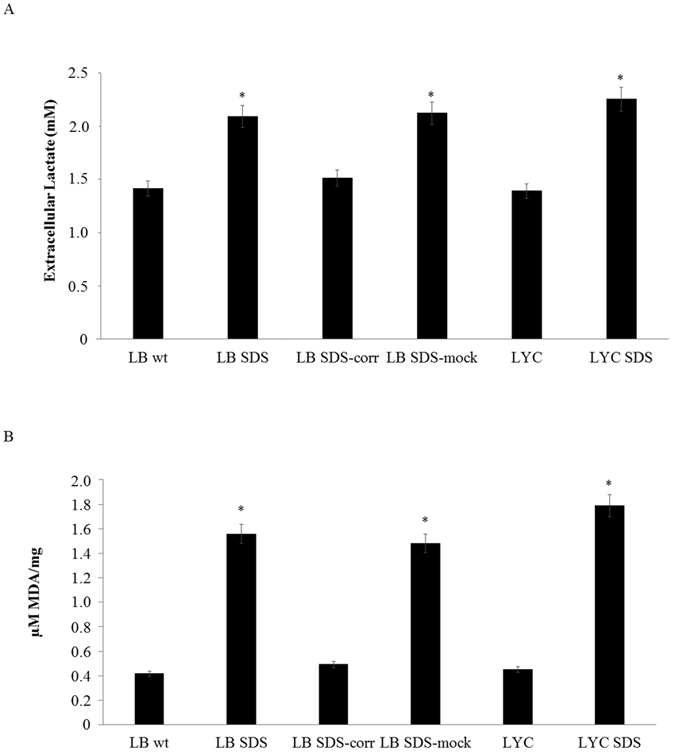
SDS cells have increased glycolysis and oxidative stress. (**A**) Extracellular lactate production is a marker for the glycolysis pathway. Lactate production was higher in the SDS lymphocyte and lymphoblast medium, suggesting an increase in the glycolysis pathway to sustain the energy request. The data are the medians of at least 3 different experiments. ^#^P < 0.005. (**B**) Malondialdehyde (MDA), a lipid peroxidation product, was utilized as a marker of oxidative stress. The MDA level was significantly increased in SDS cells compared with wt cells. The data are the medians of at least 3 different experiments. *p < 0.001. Legend: LB (lymphoblasts), LYC (lymphocytes), SDS (Shwachman-Diamond syndrome), wt (wild type), SDS-corr (Shwachman-Diamond syndrome cells transfected with the SBDS gene), SDS MOCK (Shwachman-Diamond syndrome cells transfected with empty vector).

In order to test which alternative energetic pathways might support ATP production, we investigated glycolysis, which represents the most common alternative energy source with respect to OXPHOS. As reported in Fig. 2A, we found that the lactate concentration, a marker of the glycolytic flux in the culture medium, was significantly higher in the SDS cells than in both the controls and the corrected SDS cells, confirming that the glycolysis pathway sustained the energy demand of the SDS cells.

This group of experiments demonstrates that SDS cells have increased energetic stress due to a reduced respiration ability that is caused by impaired complex IV activity and that energy supply is provided by the activation of the alternative glycolytic pathway. In particular, these defects were related to the mutated SBDS protein, as they were restored after complementation of cell lines with SBDS gene.

### Reduced oxidative phosphorylation increases ROS and malondialdehyde (MDA) production in SDS cells

A functional deficiency in OXPHOS activity is associated with increased oxidative stress products, which are increased in SBDS-silenced cells^18^. Both the lymphocytes and lymphoblast cell lines from SDS individuals had similar ATP and AMP levels as well as oxymetric parameters; therefore, in order to assess oxidative stress, we measured ROS by cytometry after staining cells with H2DCFH-DA under baseline conditions and after exposure to 100 μM H_2_O_2_ in the lymphoblast cells lines only. As expected, the H_2_O_2_ exposed SDS cells generated more ROS than controls. Although the baseline fluorescence (fluorescence arbitrary units) was only slightly increased in the SDS cells (4.2 ± 3.1) compared with the wt cells (3.4 ± 1.2), this finding was significantly higher (18.7 ± 9.4 vs 7.1 ± 2.5; p > 0.05) after H_2_O_2_ induction, thus pointing to excessive ROS production in the SDS cells under stress conditions only.

ROS attacks polyunsaturated fatty acid membranes, generating lipid peroxides^19^,^20^. As a biomarker of the lipid peroxidation process, we measured malondialdehyde, a breakdown product of lipid peroxides^21^. As shown in Fig. 2B, the MDA level was higher in the SDS cells than in the control samples (p < 0.001), thus confirming the existence of lipid peroxidation in these cells consequent to the increased oxidative stress levels.

Nevertheless, when we assessed mitochondrial morphology by electron microscopy, we did not find any relevant differences between the SDS and control cells (Supplementary Figure 3).

In conclusion, these experiments show that the SDS cells, probably in relationship with dysfunctional OXPHOS, do produce excessive ROS mainly under stressed conditions. This in turn caused lipid membrane peroxidation that, however, did not alter mitochondrial morphology. Overall, these results seem to suggest the presence of increased oxidative stress in SDS cells, which was unable to severely impair the mitochondrial structure.

### Energetic stress response pathway

In order to test which compensatory mechanisms SDS cells utilize to counteract defective energy production (energetic stress), we assessed AMP-activated protein kinase (AMPK) and the PI3K/AKT/mammalian target of rapamycin (mTOR) pathway, which are the main regulatory mechanisms activated by energy deficiency.

Under stress conditions AMPK, which is activated by AMP accumulation, antagonizes mTOR and stimulates alternative catabolic processes, such as glycolysis, which works to counteract the energy constraint. The PI3K/AKT/mTOR pathway induces cell proliferation through mitochondria and ribosome biogenesis. AKT that is phosphorylated on Thr803 by PI3K (phosphatidylinositol 3-kinase) inhibits AMPK and induces mTOR activation. mTOR in turn regulates AKT phosphorylation at Ser473, which is a site required for its maximal activation^22^,^23^,^24^,^25^,^26^. Therefore, in wild type cells, when AMPK is activated, the PI3K/AKT/mTOR pathway is normally inhibited.

**Figure 3:**
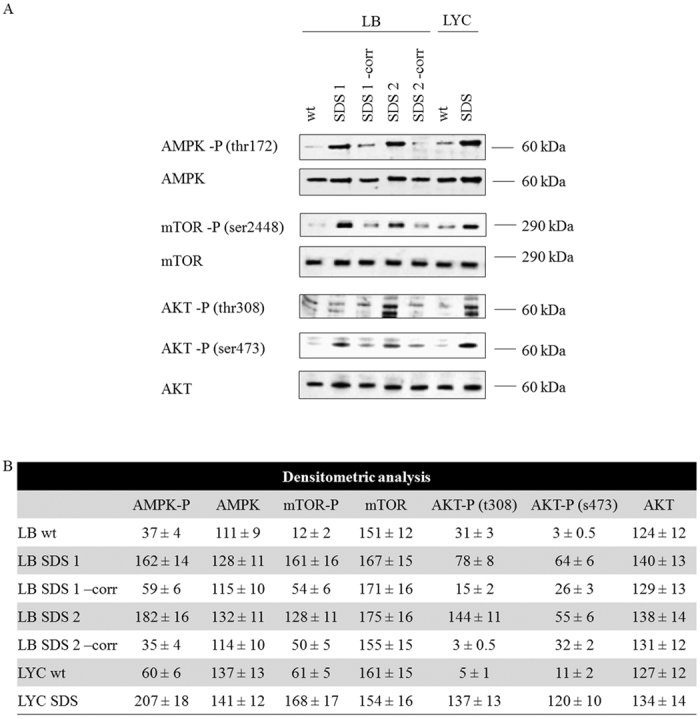
An aberrant energetic stress pathway response in SDS cells. (**A**) Western blot of the phosphorylated forms of AMPK, mTOR and AKT (Thr803 and Ser473), which showed increased activation in SDS cells. Genetic correction with the *SBDS* gene restored normal phosphorylation levels of these proteins. (**B**) Densitometric analysis of the WB signals. The data are expressed as relative optical densities.

In SDS cells, as expected, AMPK is more active than in controls, as shown by Western blot analysis of the phosphorylated form of the protein (Fig. 3). Surprisingly, we found that mTOR was not inhibited and instead it was highly phosphorylated compared with the normal and corrected controls.

Consistent with this finding, we documented that AKT was also hyper-phosphorylated at Thr803 and Ser473 in the SDS cells in comparison with wild type and corrected cells, thus confirming the hyper-activation of the whole PI3K/AKT/mTOR pathway.

In conclusion, these experiments show that SDS cells respond aberrantly to energetic stress and this response was related to the SDS protein, as PI3K/AKT/mTOR pathway hyper-activation was not seen in the corrected cells.

### SDS cells have high cytoplasmic calcium concentration levels

Ca^2+^ regulates eukaryotic protein translation^27^ and many other cellular ATP-consuming reactions; thus, it is a crucial protein synthesis and energy metabolism signalling molecule. Moreover, high [Ca^2+^]_i_ is strongly associated with ROS cytotoxicity, lipid peroxidation and OXPHOS functionality, and it inhibits complex IV activity^28^,^29^,^30^.

In resting conditions, the [Ca^2+^]_i_ was twofold (133 ± 8 nM) higher in SDS cells than in wild type (65 ± 2 nM) or corrected (64 ± 1 nM) cells (Table 1).

To investigate the cause of the increased [Ca^2+^]_i_, we assessed the ability of the ER to capture calcium from the cytoplasm and store it. Indeed, the ER is the main intracellular calcium storage site and plays an important role in Ca^2+^ homeostasis through the SERCA (calcium ATP-ase channel) activity, which transfers Ca^2+^ from the cytosol to the ER^31^. Thapsigargin (TG) irreversibly blocks the SERCA channels, causing Ca^2+^ to escape from the ER and consequently increase the [Ca^2+^]_i_^32^. Treatment with high TG doses (3 μM) induced a significant [Ca^2+^]_i_ increase in wild-type and corrected cells compared with the SDS cells, thus suggesting an impaired ability of these cells to store Ca^2+^ in the ER, which may account for the increased [Ca^2+^]_i_ (Table 1).

Even though we did not find any SERCA channel activity differences (data not shown), we speculate that the increased [Ca^2+^]_i_ may depend on the mTOR activation that we observed in the SDS cells, which is known to positively regulate calcium release via the inositol-1,4,5 triphosphate receptor^33^.

### Leucine restores the normal metabolic phenotype

**Figure 4:**
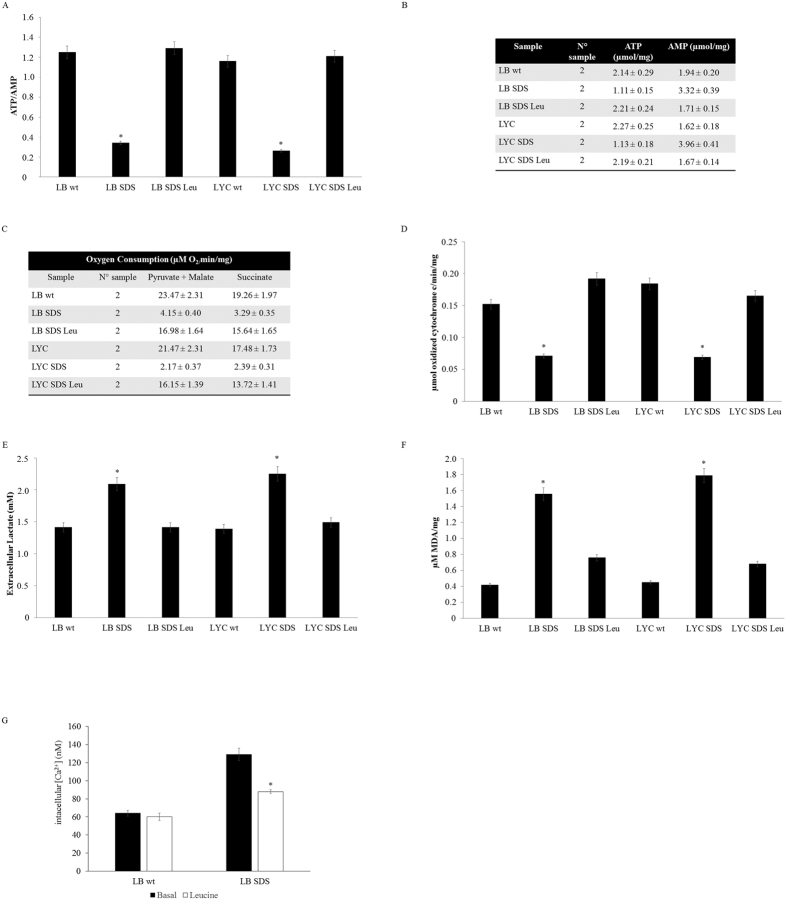
Treatment with leucine induce recovery of the respiration rate and energetic stress. ATP/AMP ratio (**A**) in SDS lymphoblast and lymphocytes was comparable to the wt cells after leucine treatment as well as ATP and AMP levels (**B**) respiration rate (**C**), complex IV activity (**D**), extracellular lactate concentration (**E**) MDA lipid peroxidation (**F**) and [Ca^2+^]_i_ (**G**).

Since leucine (Leu) is an essential amino acid known to promote proteins synthesis, even in SBDS-deficient cells^18^, we tested the biochemical effect of Leu on SDS lymphocytes and lymphoblast cells after 5 days of treatment. After Leu treatment, the complex IV functionality was restored, resulting in a respiration rate and an ATP/AMP ratio that was comparable with the controls (Fig. 4A–D) along with a reduced intracellular calcium concentration (Fig. 4G). The phenotypic reversion was also associated with a reduction in lipid peroxidation and lactate production (Fig. 4E,F). Surprisingly, treatment with N-acetyl cysteine (NAC), an antioxidant molecule that is a precursor of reduced glutathione, showed no or poor recovery of the respiratory defect, energetic distress and altered calcium homeostasis that was observed in the SDS cells (data not shown).

**Figure 5:**
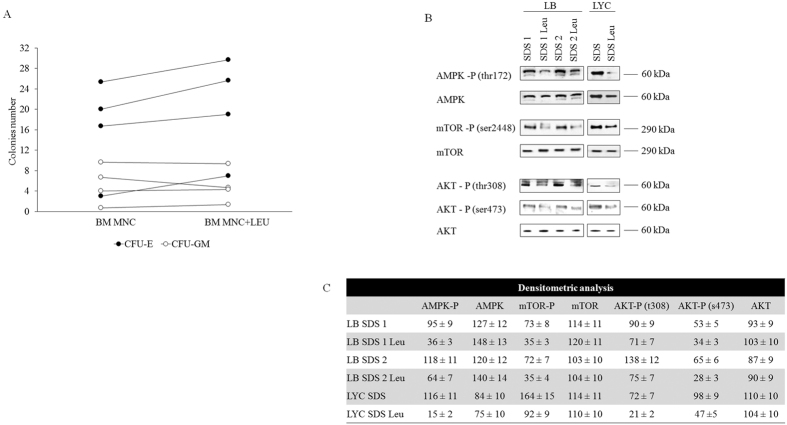
Leucine induced increased erythroid cell colonies and regulated the energetic stress biochemical pathway. (**A**) A clonogenic assay on the SDS MNCs showed increased erythroid colonies after treatment with leucine (closed circles); however, no myeloid colony induced effect was observed (open circles). (**B**) The AMPK, mTOR and AKT (Thr803 and Ser473) phosphorylation levels in SDS cells was reduced after leucine treatment and were similar to wt cell levels and was representative of respiratory and energetic stress recovery after leucine treatment. (**C**). Densitometric analysis of the WB signals. The data are expressed as relative optical densities.

We then tested the effect of Leu on marrow haematopoietic progenitor cell growth from patients with SDS, and observed a moderate increase in erythroid but not myeloid colony growth (Fig. 5A). Since Leu is a modulator of mTOR activation^34^, we studied its effects on the energetic stress pathway after chronic SDS cell treatment. As expected, the reduction in AMPK phosphorylation was a consequence of the restoration of normal OXPHOS. Interestingly, the AKT and mTOR phosphorylation levels were also reduced following the Leu treatment (Fig. 5B). It may be speculated that these effects were due to feedback regulation of the AKT/mTOR pathway that is dependent on nutrient excess^35^. Finally, as Leu restored the biochemical phenotype in the SDS cells, this molecule could be regarded as a potential tool to restore energetic metabolism in SDS cells.

## Discussion

SDS is a ribosomopathy that results from mutations affecting the expression/function of the SBDS protein, which plays a role in ribosome biogenesis and protein synthesis, two highly energy consuming processes that are finely coordinated with cellular energy production. Cellular respiration is a set of metabolic reactions and processes that converts nutrients into biochemical energy, chiefly ATP. This process is carried out by a series of protein complexes called electron transport chains that are localized in the mitochondrial inner membrane and connect to the mitochondrial intermembrane space and matrix. Impaired respiration undermines ATP production and exposes the cell to energetic and oxidative stress^17^.

In this study, we evaluate energetic metabolism in SDS cells and found that oxygen consumption was impaired when it was induced by pyruvate/malate or succinate. Consequently, ATP production was reduced and AMP accumulated, which altered the ATP/AMP ratio.

Electrons are transported through two pathways. The first, which is composed of complexes I, III and IV, transports electrons from NADH and is pyruvate/malate inducible. The other requires complexes II, III and IV and is activated by succinate, whereby it transfers the electrons from FADH_2_ and is less efficient in energy production than the first pathway. As both pyruvate/malate and succinate affect oxygen consumption to the same extent in SDS cells, we investigated the activity of the two complexes (III and IV) that are shared by the two pathways, and we demonstrated that complex IV did not work properly.

The causes of the complex IV activity impairment are not clear. The expression levels of COX5A and COX2, two subunits of complex IV that are encoded by nuclear and mitochondrial genes, respectively, were evaluated, and we observed that these two proteins were expressed at normal levels. This suggests that their synthesis was not impaired despite the ribosome biogenesis and transduction defects in the SDS cells. However, it is important to note that complex IV is composed of 32 proteins, 3 of which are encoded by mitochondrial DNA and 29 are encoded by nuclear DNA. Additionally, 11 are structural and 18 are assembling factors^36^,^37^; therefore, we cannot exclude that one of the other subunits was less expressed or not correctly folded.

Another cause may be represented by an alteration in the mitochondrial membrane, which is determined by altered electron transfer amongst complexes III and IV, or among complex IV and oxygen, resulting in impaired cytochrome c oxidase activity and increase oxidative stress^38^. However, this possibility appeared unlikely, as we measured a low level of oxidative stress and observed an apparent mitochondrial membrane integrity in the SDS cells (Supplementary Figure 3).

Notably, even though complex IV has an indirect role in ROS production, it is possible that the impairment of complex IV may be caused by upstream electron accumulation at the level of complexes I and III, which causes increased ROS production. In fact, the mitochondrial electron transport chain contains several redox centres that may directly transfer an electron to oxygen, creating ROS^39^,^40^,^41^,^42^,^43^. However, there can be other structures that may be involved in oxidative stress induction. In particular, as SDS cells are characterized by a defect in protein biogenesis, it is possible that an accumulation of the defective proteins may contribute to the unfolded protein response in the endoplasmic reticulum^44^.

Another possible cause of the complex IV impairment could be related to the reported altered calcium level in SDS cells. Intracellular calcium homeostasis plays an important role in the regulation of the biochemical pathways that modulate the response to energetic stress. In particular, Ca^2+^ has a dual effect on energetic metabolism. One effect is that the [Ca^2+^]_i_ enhances OXPHOS, which activates Krebs cycle dehydrogenases and mitochondrial substrate transporters. The other effect is that it may affect complex IV activity by competing for its cation-binding site^45^.

**Figure 6:**
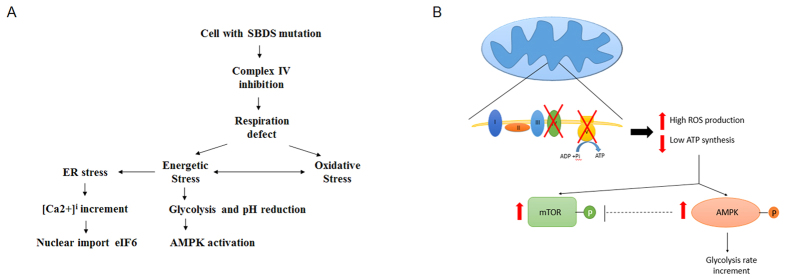
Representative scheme of the biochemical alterations in SDS cells. (**A**) The scheme represents possible interconnections among the several aspects considered in this study (see Discussion). (**B**) The scheme focuses attention on the biochemical pathways involved in the energetic status of the cell. In particular, it is possible to hypothesize that the impairment of Complex IV may be a reason for the decreased energy production and increased oxidative stress. The loss of ATP synthesis may activate the AMPK pathway, inducing an increase in the glycolysis pathway to support the cell energy demands. Moreover, the mTOR pathway also seemed to be activated, probably to improve the energy production in the mitochondria. However, in the SDS cell, these reactions led to a vicious circle, which could increase the ATP/AMP ratio impairment.

Considering these data (Fig. 6), the high [Ca^2+^]_i_ observed in SDS cells might be relevant to explain the biochemical phenotype of SDS cells. SBDS cooperates with elongation factor-like 1 (EFL1) for the release of eIF6 from the pre-60S ribosomal subunit, allowing for the formation of ribosome 80S^5^,^46^. In SDS cells, maturation of the 60S subunit is, therefore, defective and its association with the 40S subunit impaired. Moreover, the binding of eIF6 to the pre-60S subunit persists even in the cytoplasm, preventing its recycling to nucleolus^47^. Considering that the nuclear import of eIF6 is modulated by intracellular Ca^2+ ^48, we could speculate that the increased cytoplasmic calcium concentration in SDS cells attempts to balance the defective ribosome biogenesis.

Energetic stress induces changes in cellular metabolism that stimulate or inhibit a network of molecules involved in the regulation of energetic balance, such as AMPK and mTOR^49^,^50^. In the observed SDS cells, as a consequence of the energetic stress, AMPK was hyperactivated and the glycolytic pathway was stimulated. Surprisingly, we also found that the AKT/mTOR pathway was aberrantly hyper activated, as both of these proteins were hyper-phosphorylated. We speculate that mTOR hyper activation is a way through which SDS cells support the energy defect and protein synthesis, in an attempt to improve the OXPHOS activity. However, treatment of SDS cells with the mTOR antagonist, rapamycin, totally inhibited the residual OXPHOS activity, which led to decreased ROS production and a consequent energy recovery by glycolysis (data not shown). This suggests that in the altered biochemistry of the SDS cells, the increase in OXPHOS activity, which guarantees an increase in ATP production, may at the same time lead to the enhancement of the oxidative stress and a potential increase in cell damage (Fig. 6). Another potential mechanism responsible of mTOR activation in presence of an activated AMPK could be linked to autophagy activation. In ribosomopathies, increased oxidative stress induces autophagy through an mTOR-S6 kinase pathway^51^. Considering the data reported in this manuscript, we can hypothesize that the AMPK enhanced activity could be related to an impairment in the cellular energetic status, while the mTOR pathway activation might be related to a high level of oxidative stress production.

Moreover, mTOR also controls several Ca^2+^ -dependent processes. For example, it positively regulates IP3R (inositol tri-phosphate receptor)-mediated Ca^2+^ release^33^ and, in association with Akt, it participates in the regulation of mitochondrial associated endoplasmic reticulum membrane (MAM) integrity, calcium flux and energetic metabolism^52^.

Finally, an increase in glycolytic metabolism induces the accumulation of lactic acid and a decrease in the intracellular pH, resulting in the inhibition of the calcium pumps and subsequently in an increase in cytosolic [Ca^2+^]^53^ (Fig. 6).

Leucine is an essential amino acid that induces cell proliferation and protein synthesis. Ribosomopathies, such as Blackfan Diamond Anaemia and del(5q) MDS, or other pathologies with ribosomal biogenesis defects, such as Cornelia de Lange or Roberts syndrome, benefit from treatment with this amino acid^18^,^54^,^55^,^56^. Consistent with these studies, we found that leucine improved *in vitro* erythropoiesis from SDS individuals. Moreover, treatment of SDS cells with leucine restored OXPHOS and ATP synthesis, reduced the cytoplasmic calcium concentration and the AMPK and AKT/mTOR activity, which point to leucine as a potentially helpful tool in sustaining the deranged energetic metabolism and erythropoiesis of SDS patients. In particular, considering that leucine improves OXPHOS activity, partially restoring respiration, it may act as a modulator of both the AMPK and AKT/mTOR pathways by restoring their physiological roles. We can speculate that the decreased oxidative stress induced by leucine leads to a consequent inactivation of mTOR - S6 kinase pathway^51^.

Finally, it is interesting to note that energetic metabolism is a determinant for the maintenance of self-renewing stem cells. Haematopoietic stem cells (HSC) in the bone marrow are sequestered in a hypoxic microenvironment^57^. Hypoxic conditions maintain HSCs in a quiescent state, which is associated with glycolytic metabolism. During (asymmetric) self-renewal stem cell division, one cell maintains the characteristic of a stem cell with glycolytic metabolism, while the other cell moves into the blood vessels and acquires OXPHOS metabolism and differentiates. Haematopoietic stem cells are sensitive to increased oxidative stress and mitochondrial oxidative phosphorylation is the main cause of ROS. Glycolytic metabolism is therefore a determinant of the self-renewing maintenance of HSCs. To reduce mitochondrial metabolism, HSCs positively regulate AMPK while repressing the PI3K/mTOR pathway. Constitutively active AKT or mTOR signalling^58^ and high [Ca^2+^]_i_ levels^59^ cause increased proliferation and HSC impoverishment. Hence, this work may provide a new perspective in the understanding of the biochemical pathways that lead to bone marrow failure in SDS patients.

In conclusion, we report for the first time that SDS cells suffer from energetic stress and severe respiratory defects related to the faulty SBSD protein, even though SDS cells do not display a significant alteration in mitochondrial morphology. These defects are partially compensated by enhanced AMPK, glycolysis and mTOR/Akt pathway activation. A pivotal role in the maintenance of this altered metabolism could be because of altered calcium homeostasis.

## Materials and Methods

### Cell culture and treatments

Cells were obtained from the “Cell Line and DNA Biobank from Patients affected by Genetic Diseases” (G. Gaslini Institute) - Telethon Genetic Biobank Network (Project No. GTB07001)^60^. Study approval was obtained from the Institutional Review Board of all participating centres. For studies on cells and cell lines, written informed consent was obtained from patients or from relatives/guardians whenever applicable. All experiments were carried out in accordance with the approved guidelines.

SDS and wild type lymphoblast cell lines were grown at 37 °C in RPMI supplemented with 10% foetal calf serum and antibiotics. Primary lymphocytes were isolated from the PB by Ficoll gradient centrifugation and grown at 37 °C in RPMI supplemented with 10% foetal calf serum, antibiotics and phytohaemagglutinin (20 μg/ml). Leucine (600 μg/ml) was added to the cells once a day for five days. One hour after the last treatment, the cells were harvested and used for protein extracts or biochemical experiments. Thapsigargin (TG, 3 μM) was added directly to the cells in PBS buffer.

### SDS corrected cell lines

cDNA from wild-type cells was used with standard PCR to amplify the SBDS gene for 30 cycles with the following primers: sense 5′-taagatggaacaaaaactcatctcagaagaggatctgatgtcgatcttcacccccac-3′, anti-sense 5′-TTGATGGGTGTCATTCAAATTTCTCATCTC-3′. An N-terminal Myc Tag was introduced in the PCR product (sequence underlined in 5′ primer). Myc-SBDS was cloned with the TOPO® TA cloning vector system I (Invitrogen), and then subcloned into the KpnI/XhoI site of the pcDNA3.1 mammalian expression vector (Invitrogen). Identification of the recombinants was carried out by HindIII/BamHI endonuclease digestion and sequencing.

A corrected cell line was obtained by transfection of the SDS cell line with the pCDNA-Myc-SBDS vector. The transfection was carried out with lipofectamine following the manufacturer’s instruction.

The transfection efficiency was evaluated by Western blot analysis of the SBDS protein (Supplementary Figure 1).

Biochemical characterization of SDS cells was conducted on 4 lymphoblast and 4 lymphocyte cell lines. Two lymphoblast and 2 lymphocyte cell lines were used for leucine experiments, and 2 lymphoblast cell lines were used for genetic complementation.

### Cell homogenate preparation

Cultured cells were centrifuged to remove the growth medium at 1,000 g for 2 min. The pellet was suspended in phosphate buffer saline (PBS), and sonicated 2 times for 10 seconds, with a break of 30 sec in ice. Total protein levels were estimated with the Bradford method.

### ATP/AMP ratio evaluations

The ATP and AMP quantification was based on the enzyme coupling method by Bergmeyer *et al.*^61^. ATP was assayed, following NADP reduction at 340 nm. The medium contained 50 mM Tris HCl pH 8.0, 1 mM NADP, 10 mM MgCl_2_, and 5 mM glucose in 1 ml final volume. Samples were analysed spectrophotometrically before and after the addition of 4 μg of purified hexokinase/glucose-6-phosphate dehydrogenase. AMP was assayed following the NADH oxidation at 340 nm. The medium contained 50 μg of cells homogenate, 100 mM Tris-HCl (pH 8.0), 75 mM KCl, 5 mM MgCl_2_, 0,2 mM ATP, 0,5 mM phosphoenolpyruvate, 0,2 mM NADH, 10 IU adenylate kinase, 25 IU pyruvate kinase, and 15 IU of lactate dehydrogenase. Twenty micrograms of total protein was used for both assays.

### Evaluation of oxygen consumption in Shwachman cells

Oxygen consumption was measured at 25 °C in a closed chamber with an amperometric electrode (Unisense-Microrespiration, Unisense A/S, Denmark)^13^. For each experiment, 500,000 cells were used. The cells were permeabilized with 0.3 mg/ml digitonin for 1 min. Ten millimolar pyruvate plus 5 mM malate was added to stimulate the complex I, III and IV pathways. Twenty millimolar succinate was added to stimulate the complex II, III and IV pathways. Rotenone (0.1 mM) or antimycin A (0.2 mM) were used as inhibitors for the first and second pathways, respectively. Rotenone is considered a specific inhibitor of complex I, the NADH-ubiquinone oxide reductase, because it blocks the transfer of electrons from iron-sulfur centres N2 to ubiquinone. This creates a back-up of electrons within the mitochondrial matrix^62^.

Antimycin A is an antibiotic that specifically inhibits complex III, cytochrome c reductase. It acts by blocking the transfer of electrons between Cyt bH and coenzyme Q that is bound at the QN site^63^.

### Respiratory complex assay activity

For each assay, 20 μg of total protein were used. The assays were performed spectrophotometrically, as previous reported^64^.

### Extracellular lactate evaluation

The lactate concentration was assayed spectrophotometrically in the growth medium, following the reduction of NAD^+^ at 340 nm^61^. The assay medium contained 100 mM Tris HCl pH 8, 5 mM NAD^+^, 1 IU/ml of lactate dehydrogenase. Samples were analysed spectrophotometrically before and after the addition of 4 μg of purified lactate dehydrogenase. The data were normalized by the cell number.

### Cytometry and malondialdehyde evaluation in Shwachman cells

Stress induced by cell exposure to H_2_O_2_ and ROS was quantified by flow cytometry measurements after staining cells with H_2_DCFDA (2′,7′-dichlorodihydrofluorescein diacetate), according to Cuccarolo *et al.*^29^. To assess the lipid peroxidation in the Shwachman cells, the malondialdehyde (MDA) concentration was evaluated with the thiobarbituric acid reactive substances (TBARS) assay^65^ at 532 nm.

### Electron microscopy

For this analysis lymphocyte pellets from healthy donors and patients with SDS were employed. Study approval was obtained from the Institutional Review Board of all participating centres. For studies on cells and cell lines, written informed consent was obtained from patients or from relatives/guardians whenever applicable. All experiments were carried out in accordance with the approved guidelines.

Cell pellets were fixed with 2.5% glutaraldehyde/0.1 M cacodylate buffer at pH 7.6 for 1 h at room temperature. After post-fixation with 1% OsO4 in cacodylate buffer for 1 h, pellets were dehydrated in an ethanol series and embedded in Epon resin. Ultrathin sections stained with uranyl-acetate and lead citrate were observed with a Jeol Jem-1011 transmission electron microscope. Two hundred mitochondria were examined for each sample.

### Western Blot

Denaturing electrophoresis (SDS–PAGE) was performed with a Laemmli protocol, with minor modifications, in a Mini Protean III (BioRad, Hercules, CA, USA) apparatus. Twenty micrograms of total protein was used for each sample. Electrophoretically separated samples was transferred onto nitrocellulose (NC) membranes by electroblotting at 400 mA for 1 h at 4 °C. The NC membranes were blocked in 5% bovine serum albumin (BSA) overnight, then washed in 0.15% phosphate buffered saline-tween (PBS-T) and incubated with specific antibodies (Abs) (overnight at 4 °C) against phospho-AMPKα(Thr172), AMPKα, phospho-AKT_Ser473, phospho-AKT_Thr308, AKT, phospho-mTOR_Ser2448, mTOR, COX5A, COX2, ND4L, Actin and SBDS (all purchased from Cell Signalling). Secondary HPR-conjugated Ab_s_ were 1:10,000 diluted in PBS-T. Abs was revealed with an enhanced chemiluminescence detection system using ChemiDoc XRS (BioRad, Hercules, CA, USA). Densitometric analysis was performed with ChemiDoc XRS, and the density signals were comparing against actin, which was used as a housekeeping protein.

### Ca^2+^ ATPase activity assay

Ca^2+^ ATPase activity was determined from cell homogenates at 25 °C using an enzyme-coupled spectrophotometric assay, in which ATP hydrolysis is coupled to NADH oxidation^66^.

### Clonogenic assay

*In vitro* assays for erythroid colony- and burst-forming unit growth (CFU-Es and BFU-Es, respectively) were performed using methyl cellulose media (StemCell Technologies, Vancouver, Canada) complemented with cytokines, as described elsewhere^67^.

### Cytosolic [Ca^2+^]_i_ concentration calculations

Intracellular calcium 2^+^ concentrations [Ca^2+^]_i_ were measured by using the ratiometric membrane-permeant fluorescent indicator dye, Fura2/AM (Invitrogen, Life Technologies, Italy). Cells grown on 20 mm coverslips were incubated with 10 μM Fura2/AM in standard PBS buffer for 45 min at 37 °C and then washed at room temperature. The intracellular free Ca^2+^ concentration was calculated according to following equation: [Ca^2+^]_i_ = β*K*_*d*_*(R* − *R*_min_)/(*R*_max_ − R) where *R* is *E*_340_/*E*_380_; *R*_min_ is *E*_340_/*E*_380_ in zero Ca^2+^; *R*_max_ is *E*_340_/*E*_380_ in the Ca^2+^ -saturated solution; β is *E*_380_ in the zero Ca^2+^/*E*_380_ in Ca^2+^ saturated solution and *K*_*d*_ is the dissociation constant of the dye at room temperature (140 nM). To obtain the *R*_min_ and *R*_max_ values, the Ca^2+^ ionophore, ionomycin (2 nM), was added after each experiment in a zero-Ca^2+^ bath (0 Ca^2+^, 2 mM EGTA) and then the cells were perfused with a saturating Ca^2+^ solution. At the end of this procedure, 5 mM MnCl_2_ was added to the bath to quench the fluorescence of the dye and to determine the background values.

### Statistical analysis

Data were analysed with one-way ANOVA and unpaired two-tail Student’s t tests using the instat software (GraphPad Software, Inc., La Jolla, CA, USA). Data are expressed as means ± standard deviation (SD) from 3 to 5 independent determinations performed in duplicate. In the figures, the SDs are shown as error bars. An error probability with P < 0.05 was selected as the significance level.

## Additional Information

**How to cite this article**: Ravera, S. *et al.* Evaluation of energy metabolism and calcium homeostasis in cells affected by Shwachman-Diamond syndrome. *Sci. Rep.* **6**, 25441; doi: 10.1038/srep25441 (2016).

## Supplementary Material

Supplementary Information

## Figures and Tables

**Table 1:** [Ca^2+^]_i_ measured in baseline condition and after treatment with thapsigargin in WT, SDS and SDS corrected or mock lymphoblasts.

	Basal (untreat cells)	Thapsigargin
LB wt	65 ± 2	509 ± 29
LB SDS	133 ± 8	177 ± 31
LB SDS Corr.	64 ± 1	510 ± 26
LB SDS Mock	121 ± 6	178 ± 30

Calcium concentration is expressed as nanomolar (nM). Data expressed as mean ± SD of at least 3 different experiments.

## References

[b1] Dall’oca C. *et al.* Shwachman-Diamond syndrome. Musculoskelet. Surg. 96, 81–8 (2012). 22201042 10.1007/s12306-011-0174-z

[b2] Myers K. C., Davies S. M. & Shimamura A. Clinical and molecular pathophysiology of Shwachman-Diamond syndrome: an update. Hematol. Oncol. Clin. North Am. 27, 117–128, ix (2013). 23351992 10.1016/j.hoc.2012.10.003 PMC5693339

[b3] Boocock G. R. B. *et al.* Mutations in SBDS are associated with Shwachman-Diamond syndrome. Nat. Genet. 33, 97–101 (2003). 12496757 10.1038/ng1062

[b4] Zhang S., Shi M., Hui C.-C. & Rommens J. M. Loss of the mouse ortholog of the shwachman-diamond syndrome gene (Sbds) results in early embryonic lethality. Mol. Cell. Biol. 26, 6656–6663 (2006). 16914746 10.1128/MCB.00091-06 PMC1592835

[b5] Finch A. J. *et al.* Uncoupling of GTP hydrolysis from eIF6 release on the ribosome causes Shwachman-Diamond syndrome. Genes Dev. 25, 917–929 (2011). 21536732 10.1101/gad.623011 PMC3084026

[b6] Burwick N., Coats S. A., Nakamura T. & Shimamura A. Impaired ribosomal subunit association in Shwachman-Diamond syndrome. Blood 120, 5143–5152 (2012). 23115272 10.1182/blood-2012-04-420166 PMC3537309

[b7] Wong C. C., Traynor D., Basse N., Kay R. R. & Warren A. J. Defective ribosome assembly in Shwachman-Diamond syndrome. Blood 118, 4305–4312 (2011). 21803848 10.1182/blood-2011-06-353938

[b8] Orelio C. & Kuijpers T. W. Shwachman-Diamond syndrome neutrophils have altered chemoattractant-induced F-actin polymerization and polarization characteristics. Haematologica 94, 409–413 (2009). 19211642 10.3324/haematol.13733 PMC2649349

[b9] Austin K. M. *et al.* Mitotic spindle destabilization and genomic instability in Shwachman-Diamond syndrome. J. Clin. Invest. 118, 1511–1518 (2008). 18324336 10.1172/JCI33764 PMC2263145

[b10] Ball H. L. *et al.* Shwachman-Bodian Diamond syndrome is a multi-functional protein implicated in cellular stress responses. Hum. Mol. Genet. 18, 3684–3695 (2009). 19602484 10.1093/hmg/ddp316 PMC2742402

[b11] Henson A. L. *et al.* Mitochondrial function is impaired in yeast and human cellular models of Shwachman Diamond syndrome. Biochem. Biophys. Res. Commun. 437, 29–34 (2013). 23792098 10.1016/j.bbrc.2013.06.028

[b12] Ambekar C., Das B., Yeger H. & Dror Y. SBDS-deficiency results in deregulation of reactive oxygen species leading to increased cell death and decreased cell growth. Pediatr. Blood Cancer 55, 1138–1144 (2010). 20979173 10.1002/pbc.22700

[b13] Ravera S. *et al.* Mitochondrial respiratory chain Complex I defects in Fanconi anemia complementation group A. Biochimie 95, 1828–1837 (2013). 23791750 10.1016/j.biochi.2013.06.006

[b14] Kumari U., Ya Jun W., Huat Bay B. & Lyakhovich A. Evidence of mitochondrial dysfunction and impaired ROS detoxifying machinery in Fanconi anemia cells. Oncogene 33, 165–172 (2014). 23318445 10.1038/onc.2012.583

[b15] Cappelli E. *et al.* Mitochondrial respiratory complex I defects in Fanconi anemia. Trends Mol. Med. 19, 513–514 (2013). 23932594 10.1016/j.molmed.2013.07.008

[b16] Boyer P. D. A research journey with ATP synthase. J Biol Chem 277, 39045–39061 (2002). 12181328 10.1074/jbc.X200001200

[b17] Bonnard C. *et al.* Mitochondrial dysfunction results from oxidative stress in the skeletal muscle of diet-induced insulin-resistant mice. J. Clin. Invest. 118, 789–800 (2008). 18188455 10.1172/JCI32601 PMC2176186

[b18] Sen S. *et al.* The ribosome-related protein, SBDS, is critical for normal erythropoiesis. Blood 118, 6407–6417 (2011). 21963601 10.1182/blood-2011-02-335190

[b19] Halliwell B. & Gutteridge J. M. Role of free radicals and catalytic metal ions in human disease: an overview. Methods Enzymol. 186, 1–85 (1990). 2172697 10.1016/0076-6879(90)86093-b

[b20] Kehrer J. P. Free radicals as mediators of tissue injury and disease. Crit. Rev. Toxicol. 23, 21–48 (1993). 8471159 10.3109/10408449309104073

[b21] Ayala A., Muñoz M. F. & Argüelles S. Lipid peroxidation: production, metabolism, and signaling mechanisms of malondialdehyde and 4-hydroxy-2-nonenal. Oxid. Med. Cell. Longev. 2014, 360438 (2014). 24999379 10.1155/2014/360438 PMC4066722

[b22] Morita M. *et al.* mTORC1 controls mitochondrial activity and biogenesis through 4E-BP-dependent translational regulation. Cell Metab. 18, 698–711 (2013). 24206664 10.1016/j.cmet.2013.10.001

[b23] Iadevaia V., Liu R. & Proud C. G. mTORC1 signaling controls multiple steps in ribosome biogenesis. Semin. Cell Dev. Biol. 36, 113–120 (2014). 25148809 10.1016/j.semcdb.2014.08.004

[b24] Morita M. *et al.* mTOR coordinates protein synthesis, mitochondrial activity and proliferation. Cell Cycle 14, 473–480 (2015). 25590164 10.4161/15384101.2014.991572 PMC4615141

[b25] Bracho-Valdés I. *et al.* mTORC1- and mTORC2-interacting proteins keep their multifunctional partners focused. IUBMB Life 63, 896–914 (2011). 21905202 10.1002/iub.558

[b26] Goo C. K. *et al.* PTEN/Akt signaling controls mitochondrial respiratory capacity through 4E-BP1. PLos One 7, e45806 (2012). 23049865 10.1371/journal.pone.0045806 PMC3458951

[b27] Behnen P. *et al.* Calcium-dependent interaction of calmodulin with human 80S ribosomes and polyribosomes. Biochemistry 51, 6718–6727 (2012). 22856685 10.1021/bi3005939

[b28] Pérez-De La Cruz V. *et al.* Cytoplasmic calcium mediates oxidative damage in an excitotoxic/energetic deficit synergic model in rats. Eur. J. Neurosci. 27, 1075–1085 (2008). 18364032 10.1111/j.1460-9568.2008.06088.x

[b29] Cuccarolo P., Viaggi S. & Degan P. New insights into redox response modulation in Fanconi’s anemia cells by hydrogen peroxide and glutathione depletors. FEBS J. 279, 2479–2494 (2012). 22578062 10.1111/j.1742-4658.2012.08629.x

[b30] Vygodina T., Kirichenko A. & Konstantinov A. A. Direct regulation of cytochrome c oxidase by calcium ions. PLos One 8, e74436 (2013). 24058566 10.1371/journal.pone.0074436 PMC3769247

[b31] Mekahli D., Bultynck G., Parys J. B., De Smedt H. & Missiaen L. Endoplasmic-reticulum calcium depletion and disease. Cold Spring Harb. Perspect. Biol. 3 (2011). 10.1101/cshperspect.a004317 PMC3098671 21441595

[b32] Gilabert J. A., Bakowski D. & Parekh A. B. Energized mitochondria increase the dynamic range over which inositol 1,4,5-trisphosphate activates store-operated calcium influx. EMBO J. 20, 2672–2679 (2001). 11387202 10.1093/emboj/20.11.2672 PMC125482

[b33] Frégeau M.-O., Régimbald-Dumas Y. & Guillemette G. Positive regulation of inositol 1,4,5-trisphosphate-induced Ca^2+^ release by mammalian target of rapamycin (mTOR) in RINm5F cells. J. Cell. Biochem. 112, 723–733 (2011). 21268094 10.1002/jcb.23006

[b34] Kim S. G., Buel G. R. & Blenis J. Nutrient regulation of the mTOR Complex 1 signaling pathway. Mol. Cells 35, 463–473 (2013). 23694989 10.1007/s10059-013-0138-2 PMC3887879

[b35] Manning B. D. Balancing Akt with S6K: implications for both metabolic diseases and tumorigenesis. J. Cell Biol. 167, 399–403 (2004). 15533996 10.1083/jcb.200408161 PMC2172491

[b36] Balsa E. *et al.* NDUFA4 is a subunit of complex IV of the mammalian electron transport chain. Cell Metab. 16, 378–386 (2012). 22902835 10.1016/j.cmet.2012.07.015

[b37] Tsukihara T. *et al.* Structures of metal sites of oxidized bovine heart cytochrome c oxidase at 2.8A. Science (80-.). 269, 1069–1074 (1995). 10.1126/science.7652554 7652554

[b38] Benard G. *et al.* Mitochondrial bioenergetics and structural network organization. J. Cell Sci. 120, 838–848 (2007). 17298981 10.1242/jcs.03381

[b39] Cadenas E. & Davies K. J. A. Mitochondrial free radical generation, oxidative stress, and aging11This article is dedicated to the memory of our dear friend, colleague, and mentor Lars Ernster (1920–1998), in gratitude for all he gave to us. Free Radic. Biol. Med. 29, 222–230 (2000). 11035250 10.1016/s0891-5849(00)00317-8

[b40] Babcock G. T. & Wikström M. Oxygen activation and the conservation of energy in cell respiration. Nature 356, 301–309 (1992). 1312679 10.1038/356301a0

[b41] Babcock G. T. & Varotsis C. Discrete steps in dioxygen activation–the cytochrome oxidase/O2 reaction. J. Bioenerg. Biomembr. 25, 71–80 (1993). 8389752 10.1007/BF00762849

[b42] Varotsis C., Zhang Y., Appelman E. H. & Babcock G. T. Resolution of the reaction sequence during the reduction of O2 by cytochrome oxidase. Proc. Natl. Acad. Sci. USA 90, 237–241 (1993). 8380495 10.1073/pnas.90.1.237 PMC45635

[b43] Dawson T. L., Gores G. J., Nieminen A. L., Herman B. & Lemasters J. J. Mitochondria as a source of reactive oxygen species during reductive stress in rat hepatocytes. Am. J. Physiol. 264, C961–C967 (1993). 8386454 10.1152/ajpcell.1993.264.4.C961

[b44] Bravo R. *et al.* Endoplasmic reticulum and the unfolded protein response: dynamics and metabolic integration. Int. Rev. Cell Mol. Biol. 301, 215–290 (2013). 23317820 10.1016/B978-0-12-407704-1.00005-1 PMC3666557

[b45] Vygodina T. V., Kirichenko A. & Konstantinov A. A. Cation binding site of cytochrome c oxidase: progress report. Biochim. Biophys. Acta 1837, 1188–95 (2014). 24607866 10.1016/j.bbabio.2014.02.025

[b46] Brina D., Miluzio A., Ricciardi S. & Biffo S. eIF6 anti-association activity is required for ribosome biogenesis, translational control and tumor progression. Biochim. Biophys. Acta, 10.1016/j.bbagrm.2014.09.010 (2014). 25252159

[b47] Menne T. F. *et al.* The Shwachman-Bodian-Diamond syndrome protein mediates translational activation of ribosomes in yeast. Nat. Genet. 39, 486–495 (2007). 17353896 10.1038/ng1994

[b48] Biswas A. *et al.* Opposing action of casein kinase 1 and calcineurin in nucleo-cytoplasmic shuttling of mammalian translation initiation factor eIF6. J. Biol. Chem. 286, 3129–3138 (2011). 21084295 10.1074/jbc.M110.188565 PMC3024805

[b49] Fähling M. Cellular oxygen sensing, signalling and how to survive translational arrest in hypoxia. Acta Physiol. (Oxf). 195, 205–230 (2009). 18764866 10.1111/j.1748-1716.2008.01894.x

[b50] Bhaskar P. T. & Hay N. The two TORCs and Akt. Dev. Cell 12, 487–502 (2007). 17419990 10.1016/j.devcel.2007.03.020

[b51] Heijnen H. F. *et al.* Ribosomal protein mutations induce autophagy through S6 kinase inhibition of the insulin pathway. PLos Genet. 10, e1004371 (2014). 24875531 10.1371/journal.pgen.1004371 PMC4038485

[b52] Betz C. *et al.* Feature Article: mTOR complex 2-Akt signaling at mitochondria-associated endoplasmic reticulum membranes (MAM) regulates mitochondrial physiology. Proc. Natl. Acad. Sci. USA 110, 12526–12534 (2013). 23852728 10.1073/pnas.1302455110 PMC3732980

[b53] Kadenbach B., Arnold S., Lee I. & Hüttemann M. The possible role of cytochrome c oxidase in stress-induced apoptosis and degenerative diseases. Biochim. Biophys. Acta 1655, 400–8 (2004). 15100056 10.1016/j.bbabio.2003.06.005

[b54] Xu B., Lee K. K., Zhang L. & Gerton J. L. Stimulation of mTORC1 with L-leucine rescues defects associated with Roberts syndrome. PLos Genet. 9, e1003857 (2013). 24098154 10.1371/journal.pgen.1003857 PMC3789817

[b55] Xu B., Sowa N., Cardenas M. E. & Gerton J. L. L-leucine partially rescues translational and developmental defects associated with zebrafish models of Cornelia de Lange syndrome. Hum. Mol. Genet. 24, 1540–1555 (2015). 25378554 10.1093/hmg/ddu565 PMC4351377

[b56] Payne E. M. *et al.* L-Leucine improves the anemia and developmental defects associated with Diamond-Blackfan anemia and del(5q) MDS by activating the mTOR pathway. Blood 120, 2214–2224 (2012). 22734070 10.1182/blood-2011-10-382986 PMC3447780

[b57] Zhang C. C. & Sadek H. A. Hypoxia and metabolic properties of hematopoietic stem cells. Antioxid. Redox Signal. 20, 1891–1901 (2014). 23621582 10.1089/ars.2012.5019 PMC3967354

[b58] Hoshii T., Matsuda S. & Hirao A. Pleiotropic roles of mTOR complexes in haemato-lymphopoiesis and leukemogenesis. J. Biochem. 156, 73–83 (2014). 24962700 10.1093/jb/mvu037

[b59] Paredes-Gamero E. J., Barbosa C. M. V. & Ferreira A. T. Calcium signaling as a regulator of hematopoiesis. Front. Biosci. (Elite Ed). 4, 1375–1384 (2012). 10.2741/467 22201962

[b60] Filocamo M. *et al.* Cell Line and DNA Biobank From Patients Affected by Genetic Diseases. Open J. Bioresour. 1, e2 (2014).

[b61] Bergmeyer H. U. In Method of Enzymatic Analysis 3rd edn, Vol. 2 (eds Chemie Verlag) Ch.2 Reagents for Enzymatic Analysis 248–249 (Weinheim – Basel 1983).

[b62] Lümmen P. Complex I inhibitors as insecticides and acaricides1Dedicated to the memory of Dr. Gerhard Salbeck.1. Biochim. Biophys. Acta - Bioenerg. 1364, 287–296 (1998). 10.1016/s0005-2728(98)00034-6 9593947

[b63] Lai B. *et al.* Inhibition of Qi site of mitochondrial complex III with antimycin A decreases persistent and transient sodium currents via reactive oxygen species and protein kinase C in rat hippocampal CA1 cells. Exp. Neurol. 194, 484–494 (2005). 16022873 10.1016/j.expneurol.2005.03.005

[b64] Bartolucci M. *et al.* Functional Expression of Electron Transport Chain and FoF 1-ATP Synthase in Optic Nerve Myelin Sheath. Neurochem. Res. 10.1007/s11064-015-1712-0 (2015). 26334391

[b65] el-Saadani M. *et al.* A spectrophotometric assay for lipid peroxides in serum lipoproteins using a commercially available reagent. J. Lipid Res. 30, 627–630 (1989). 2754343

[b66] Usai C. *et al.* Dysregulated Ca^2+^ homeostasis in Fanconi anemia cells. Sci. Rep. 5, 8088 (2015). 25627108 10.1038/srep08088 PMC4308711

[b67] Dufour C. *et al.* TNF-alpha and IFN-gamma are overexpressed in the bone marrow of Fanconi anemia patients and TNF-alpha suppresses erythropoiesis *in vitro*. Blood 102, 2053–2059 (2003). 12750172 10.1182/blood-2003-01-0114

