# Supplementary material for: Evaluation of energy metabolism and calcium homeostasis in cells affected by Shwachman-Diamond syndrome
Source: Sci Rep. 2016 May 5;6:25441. doi: 10.1038/srep25441 (PMC4857091; doi:10.1038/srep25441)

Evaluation of energy metabolism and calcium homeostasis in cells affected by Shwachman-Diamond syndrome.

Silvia Ravera[1], Carlo Dufour[2], Simone Cesaro[3], Roberta Bottega [4] , Michela Faleschini [4],Paola Cuccarolo [5], Fabio Corsolini [6], Cesare Usai [7], Marta Columbaro [8], Marco Cipolli [9], Anna Savoia [4], Paolo Degan [5], Enrico Cappelli [2]

[1]DIFAR-Biochemistry Lab., Department of Pharmacy, University of Genova, 16132 Genova, Italy; [2] Hematology Unit, Istituto Giannina Gaslini, 16148 Genova, Italy; [3] Pediatric Onco-Hematology Hospital of Verona, Verona, Italy; [4] Institute for Maternal and Child Health – IRCCS Burlo Garofolo, Trieste, Italy;[5] S. C. Mutagenesis, IRCCS AOU San Martino – IST (Istituto Nazionale per la Ricerca sul Cancro), CBA Torre A2, 16123 Genova, Italy; [6] Centro Diagnostica Genetica e Biochimica Malattie Metaboliche, Istituto Giannina Gaslini, 16148 Genova, Italy; [7]Institute of Biophysics, National Research Council, 16149 Genova, Italy; [8] SC Laboratory of Musculoskeletal Cell Biology, IOR, Bologna, Italy; [9] Cystic Fibrosis Centre, Azienda Ospedaliera Universitaria, Piazzale Stefani, 1-37126 Verona, Italy.

Corresponding Author:

Enrico Cappelli - Hematology Unit, Istituto Giannina Gaslini, 16148 Genova, Italy. Tel. +3901056362693 – Fax +39010386204 – e-mail: enricocappelli@ospedale-gaslini.ge.it

Supplemental Figure 1. WB of SBDS protein show the transfection efficiency in SDS corrected cells.

A. WB analysis against SBDS protein **B.** Densitiometric analysis of the WB signal, reported as Relative Optical Density, normalized versus actin. Both data confirm that SBDS is expressed only in wt sample and in the SDS-corrected samples. Data are the media of at least 3 different experiments.

Legend: LB (lymphoblasts), SDS (shwachman-diamond syndrome), wt (wild type), SDS-corr (shwachman-diamond syndrome transfected with SBDS gene), SDS-mock (shwachman-diamond syndrome transfected with empty vector).

A

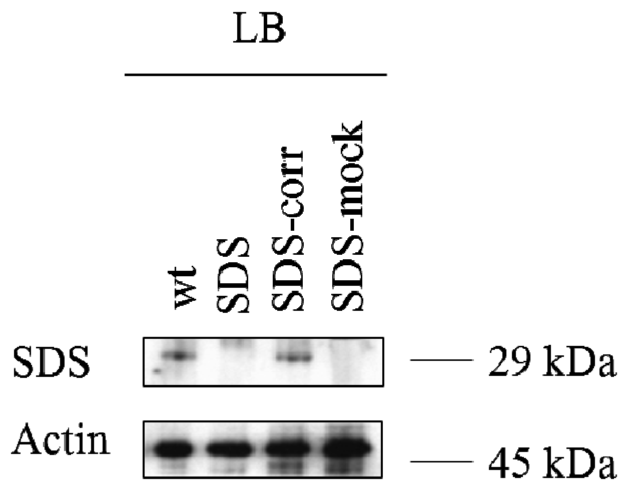

B

Densitometric analysis		
	SBDS	Actin
wt	75 ± 7	209 ± 20
SDS	0.5 ± 0.1	208 ± 20
SDS-corr	71 ± 7	209 ± 20
SDS-mock	7 ± 1	229 ±20

Supplemental Figure 2. Complexes I, II and III activity was comparable in SDS and wt cells. The graphs show that the activities of Complexes I (**A**), II (**B**) and III (**C**) are present in all sample, without significant difference. Data are the media of at least 3 different experiments.

Legend: LB (lymphoblasts), SDS (shwachman-diamond syndrome), wt (wild type), SDS-corr (shwachman-diamond syndrome transfected with SBDS gene), SDS-mock (shwachman-diamond syndrome transfected with empty vector).

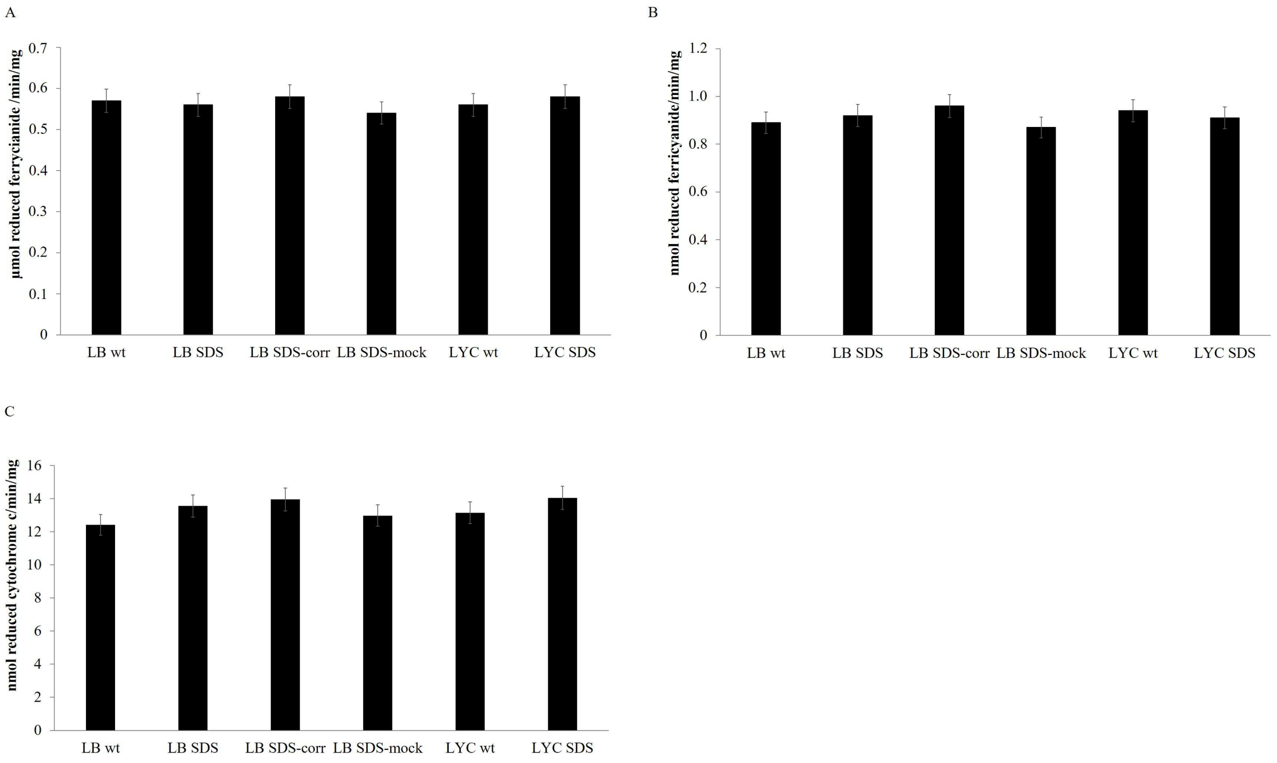

Supplemental Figure 3. Morphological features of mitochondria in SDS and healthy control lymphocytes. Representative transmission electron microscopy images of morphologically normal cells and mitochondria (arrows) in control (**a, c**) and SDS (**b, d)** lymphocytes. Scale bar, 1μm.

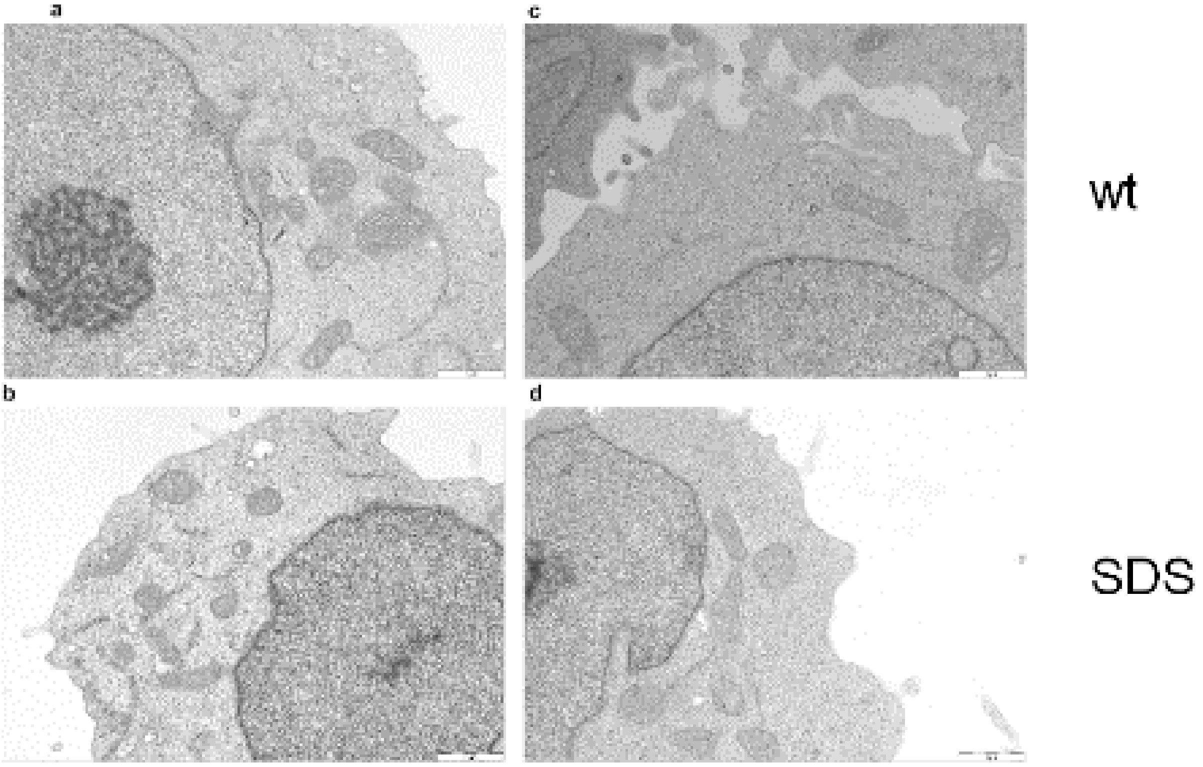

Supplement: Supplementary Information [file srep25441-s1.pdf]
